# Long-Lasting Effects of Early-Life Antibiotic Treatment and Routine Animal Handling on Gut Microbiota Composition and Immune System in Pigs

**DOI:** 10.1371/journal.pone.0116523

**Published:** 2015-02-06

**Authors:** Dirkjan Schokker, Jing Zhang, Stéphanie A. Vastenhouw, Hans G. H. J. Heilig, Hauke Smidt, Johanna M. J. Rebel, Mari A. Smits

**Affiliations:** 1 Wageningen UR Livestock Research, P.O. box 65, 8200 AB Lelystad, The Netherlands; 2 Laboratory of Microbiology, Wageningen University, Dreijenplein 10, 6703 HB Wageningen, The Netherlands; 3 Central Veterinary Institute, P.O. box 65, 8200 AB Lelystad, The Netherlands; German Institute of Human Nutrition Potsdam-Rehbrücke, GERMANY

## Abstract

**Background:**

In intensive pig husbandry systems, antibiotics are frequently administrated during early life stages to prevent respiratory and gastro-intestinal tract infections, often in combination with stressful handlings. The immediate effects of these treatments on microbial colonization and immune development have been described recently. Here we studied whether the early life administration of antibiotics has long-lasting effects on the pig’s intestinal microbial community and on gut functionality.

**Methodology/Principal Findings:**

To investigate the long-lasting effect of early-life treatment, piglets were divided into three different groups receiving the following treatments: 1) no antibiotics and no stress, 2) antibiotics and no stress, and 3) antibiotics and stress. All treatments were applied at day four after birth. Sampling of jejunal content for community scale microbiota analysis, and jejunal and ileal tissue for genome-wide transcription profiling, was performed at day 55 (~8 weeks) and day 176 (~25 weeks) after birth. Antibiotic treatment in combination with or without exposure to stress was found to have long-lasting effects on host intestinal gene expression involved in a multitude of processes, including immune related processes.

**Conclusions/Significance:**

The results obtained in this study indicate that early life (day 4 after birth) perturbations have long-lasting effects on the gut system, both in gene expression (day 55) as well as on microbiota composition (day 176). At day 55 high variance was observed in the microbiota data, but no significant differences between treatment groups, which is most probably due to the newly acquired microbiota during and right after weaning (day 28). Based on the observed difference in gene expression at day 55, it is hypothesized that due to the difference in immune programming during early life, the systems respond differently to the post-weaning newly acquired microbiota. As a consequence, the gut systems of the treatment groups develop into different homeostasis.

## Introduction

The efficient uptake of nutrients and maintenance of immune homeostasis are major prerequisites for a healthy pig gut. Both characteristics are influenced by so far unknown host genetic factors, components in the animal feed, and the composition and diversity of the microbiota residing in the lumen as well as associated with the mucosal surfaces of the gut. During life, pigs eat animal feeds that differ significantly in composition. Milk, for example, is an important feed constituent during early life, whereas dietary fibres become important at older age. It is known that such dietary changes greatly affect the composition and diversity of the microbiota in the gut [1,2]. Although different feeds display different effects, usually in the period between weaning and slaughter pigs get one feed at the farm.

Immediately after birth, the gut of piglets is colonized by microbiota derived from the sow and the environment. From studies in model organisms (rodents), but also in pigs, it has become clear that this primary colonization is important for the right development and programming of the animal’s local and systemic immune system [3,4]. Since at this stage the necessary regulatory and epigenetic processes underlying gut immune homeostasis have probably not been fully programmed yet, the composition and diversity of the colonizing microbiota is highly susceptible to environmental variations [5–9]. Experimentally induced changes in early life environmental factors have been associated with variations in the microbial composition at later ages [10], with variations in immune characteristics [11], and with differences in the susceptibility to immunological disorders [12–15].

In modern pig husbandry systems, piglets experience a variety of environmental factors that change in time and/or intensity, for example during periods of weaning, transport, and mixing of the animals, by changes in temperature, feedstuffs, the use of medicines (antibiotics, vaccines), and by the exposure to (pathogenic) micro-organisms. The short-term and long-term effects of such environmental changes on physiologic and immunologic parameters have mainly been studied in several-weeks-old piglets. Some publications have reported on the short-term effects of experimentally induced environmental variations during the early life stages of piglets [6]. In a previous paper we reported on the short-term effects of an antibiotic and stress treatment at 4 days after birth as experienced by piglets under normal intensive husbandry conditions [16]. So far, not much is known about the long-term effects of early life environmental factors as experienced by piglets under normal intensive husbandry conditions. To this end it should be noted that one recent study looked at short and long-term effects of amoxicillin treatment of sows, focusing on the microbial and physiological characteristics of piglets [17].

The objective of this study was to investigate the effects of the exposure of piglets to an antibiotic, alone or in combination with stressful management practices at day four after birth. Such treatments belong to common practices in intensive pig farming systems. The specific objective was to identify and characterize changes in the composition and diversity of the microbiota in the lumen of the gut and the concomitant (immunological) effects in intestinal tissue at 51 and 172 days after the environmental intervention. To this end, we used community-scale analysis of gut lumen microbiota and genome-wide transcriptome profiling of intestinal tissue.

## Material and Methods

### Design

**Fig 1:**
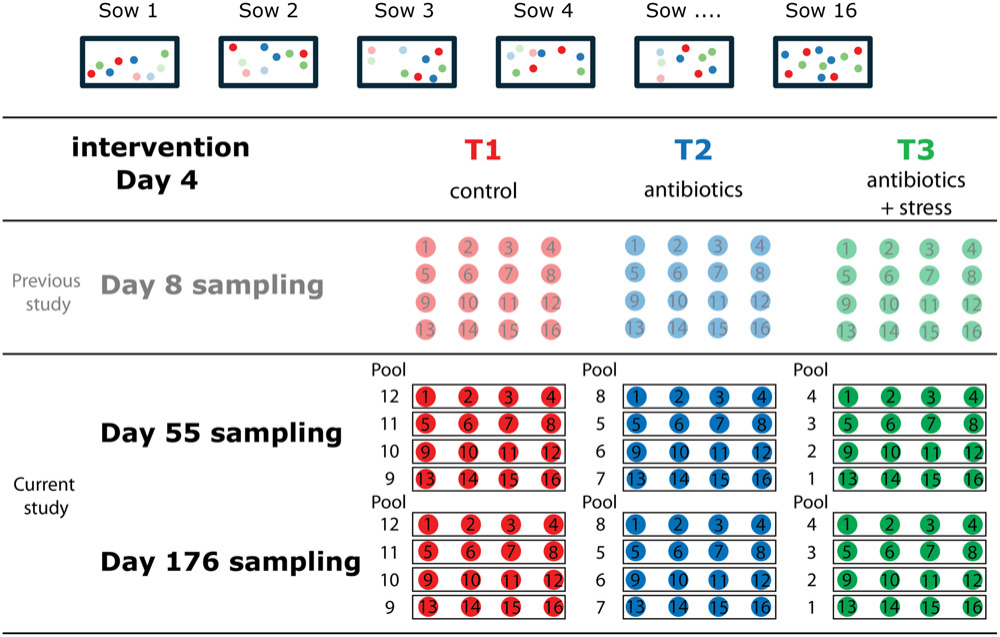
Schematic representation of the experimental design.

This study forms part of a larger study, the first part of which has been described previously [16]. In short, piglets of 16 sows (TOPIGS20 (GY x NL)) were divided into three treatment groups (T1, T2, and T3). Each litter of each individual sow contained four T1, four T2, and four T3 piglets (Fig. 1). Pools were made both for microbiota and host gene expression analyses. Pools consisted of four piglets of different litters, and in total four pools were made for each time point and each treatment. For microbiota and gene expression analyses pools consisted of the same piglets. Littermates stayed with their sow before weaning independent of treatment, and after weaning they were mixed independent of treatment. Treatment T1 consisted of no disturbance and piglets were only handled at the time of drawing blood at day 8, 55, and 176. Piglets of treatment group T2 were injected subcutaneously in the neck with 0,1 ml Tulathromycine (dosage was 2.5 mg/kg [1mL/40 kg] body weight) at day 4 after birth. Piglets of treatment group T3 were injected with 0,1 ml Tulathromycine at day 4 after birth and received the standard management procedures (i.e. docking, clipping and weighing) used at that particular farm (VIC Sterksel, The Netherlands). After weaning (from day 28) pigs were divided over different pens independent of treatment, i.e. T1, T2, and T3 animals were still together within one pen, each pen contained up to six piglets. Furthermore these animals were all housed in the same environment (stable/pen). Animals were sacrificed at day 55 or 176 by injection of 0,5–1 ml Euthasol (20% sodium pentobarbital; 200 mg/ml) intravenously. Intestinal tissues (jejunum and ileum) were taken and immediately frozen in liquid nitrogen. From the same or adjacent locations luminal contents and mucosal scrapings were taken, rinsed (with PBS) and frozen in liquid nitrogen as well.

### Ethics Statement

This animal experiment was approved by the institutional animal experiment committee “Dier Experimenten Commissie (DEC) Lelystad” (2011077.b), in accordance with the Dutch regulations on animal experiments.

### Microbiota Analysis

Jejunal contents of four piglets within each treatment were pooled and mixed in equal amounts. Microbiota profiles were only determined for jejunal contents and not ileal contents because not all piglets had actual ileal content at the time of sampling, and therefore it was not possible to determine the microbiota. Microbial DNA was extracted from 250 mg of the pooled mixture using a faecal DNA extraction protocol adapted from Yu and Morrison [18], as previously described by Salonen *et al*. [19]. After extraction of microbial DNA, the microbial composition was detected by the Pig Intestinal Tract Chip (PITChip) version 2.0, which is a phylogenetic microarray with 3,299 oligonucleotides based on 16S rRNA gene sequences of 781 porcine intestinal microbial phylotypes, designed according to the same principles previously described for the Human Intestinal Tract Chip (HITChip)[20] and PITChip version 1.0 [21,22]. The protocol for hybridization and analysis of the generated data was performed essentially as described before for the HITChip [20]. Briefly, the bacterial 16S rRNA gene was amplified using the primers T7prom-Bact-27-for (5´-TGAATTGTAATACGACTCACTATAGGGGTTTGATCCTGGCTCAG-3´) and Uni-1492-rev (5´-CGGCTACCTTGTTACGAC-3´) [20,23]. The PCR products were transcribed into RNA and the purified resultant RNA was coupled with CyDye prior to fragmentation and hybridization to the array. Microarray images were processed using Agilent’s Feature Extraction Software version 9.5 (http://www.agilent.com). Data was retrieved from the MySQL (version 5.1) database as described previously [20] and pre-processed using the R (Rx64 2.12.2) microbiome package (http://microbiome.github.com/), settings on default. Multivariate analysis was applied for PITChip data interpretation. In order to relate changes in total microbial composition to environmental variables, redundancy analysis (RDA) was used as implemented in the CANOCO 4.5 software package (Biometris, Wageningen, The Netherlands) [24]. Treatment classes were introduced as environmental (explanatory) variables. The relative contribution for 151 genus-level phylogenetic groups targeted by the PITChip were used as response variables. RDA was performed focusing on inter-sample correlation, and the Monte Carlo Permutation test was applied [25] to decide whether treatment had statistically significant influence on the microbial composition. The unrestricted permutation option (since the experiment had a randomized design) that yields completely random permutations was employed [26]. Treatment was considered to significantly affect microbial composition with p-values < 0.05. Triplot diagrams were generated using CanoDraw for Windows. To test the variation of an individual microbial group between treatments we performed a Mann-Whitney-Wilcoxon signed rank test in R (version 2.14.0) with multiple testing correction (Benjamini Hochberg). The (raw) data is available upon request by contacting one of the authors.

### Microarray Analysis

**RNA Extraction Tissue**. Total RNA was extracted from 50 to 100 mg tissue of pooled samples of jejunum and ileum. The jejunum and ileum samples were homogenised using the TisuPrep Homogenizer Omni TP TH220P) in TRizol reagent (Life Technologies) as recommended by the manufacturer with minor modifications. The homogenised tissue samples were dissolved in 5ml of TRizol reagent. After centrifugation the supernatant was transferred to a fresh tube. Subsequently a phase separation with chloroform was performed as described by the manufacturer. The RNA was precipitated and dissolved and quantified by absorbance measurements at 260 nm.

Pools were made and QC was performed with the Agilent Bioanalyzer.

**Labelling, Hybridization, Scanning and Feature Extraction**. Labelling, hybridization and washing was done as recommended by Agilent Technologies using the One-Color Microarray-Based Gene Expression Analysis Low input Quick Amp Labelling. The input for labelling was 10 ng of total RNA and 600 ng of labelled cRNA was used for hybridization on an 8 pack array. Hybridization was done in the G2545A hybridization oven (Agilent Technologies) at 65°C with rotation speed 10 rpm for 17 hours, after which the arrays were washed.

The arrays where scanned using the DNA microarray scanner with Surescan high resolution Technology (Agilent Technologies), with resolution of 5μm, 16 bits and PMT of 100%. Feature extraction was performed using protocol 10.7.3.1 (v10.7) for one colour gene expression.

**Data Loading and Statistical Analysis**. The files generated by the feature extraction software were loaded in GeneSpring GX 12 (also available in GEO, accession number; GSE53170, platform; GPL18045). After log2-transformation and quantile normalization, quality control was performed and 3 samples were removed; ileum ‘T2 day 55 pool 8’, jejunum ‘T2 day 176 pool 7’ and ileum ‘T3 day 176 pool 1’. The remaining 45 samples were analysed by principle component analysis, which showed clustering of samples of similar treatments and no clustering of tissue samples.

The next step was filtering on expression levels in which only the (20–100)^th^ percentile was included and (multiple) probes were mapped to genes if possible. To calculate whether the difference between treatments was significant a 2-way ANOVA with multiple testing correction (Benjamini-Hochberg) was performed within GeneSpring, where we compared the following groups for both jejunum and ileum: T3 vs. T1, T2 vs. T1, and T3 vs. T2. All probes/genes, which were significant under p_cor_ < 0.05 and Fold Change > |1.5| in one of the six comparisons, were taken for further functional and enrichment analyses.

**Functional Annotation Clustering**. Functional annotation clustering analyses were performed with Database for Annotation, Visualization and Integrated Discovery (DAVID, version 6.7 [27,28]). With DAVID Functional Annotation Clustering, enrichment is calculated for annotation terms of interest across multiple databases and ranked accordingly to their enrichment score (ES). When a term has an ES above 1.3 it suggests that this process/these processes are dominant within the tissue. For each comparison, i.e. T3 vs. T1, T2 vs. T1, and T3 vs. T2, analyses were performed with up- and down-regulated genes.

The DAVID tool is using GO-annotation terminology, this GO annotation terminology is frequently based on the first discoveries of functions of genes. This, however, does not automatically imply that such a gene has the same function in other tissues, although it is likely that it has similar or related functions in other tissues.

**Gene Set Enrichment Analysis (GSEA)**. GSEA [29,30] was performed separately for ileum and jejunum. We loaded the normalized intensity values of all annotated genes per treatment and time-point. The following comparisons were analysed for both day 55 and 176, T3 vs. T1, T2 vs. T1, and T3 vs. T2. The following settings were different from the default settings, permutations were performed on the gene set, chip platform was set to gene symbol. Six gene set databases (v3.0) were loaded for analysis, namely three Gene Ontology related gene sets, biological processes, molecular function and cellular component, and three pathway related gene sets, BioCarta, Reactome and KEGG.

## Results

### Microbiota analyses

**Fig 2:**
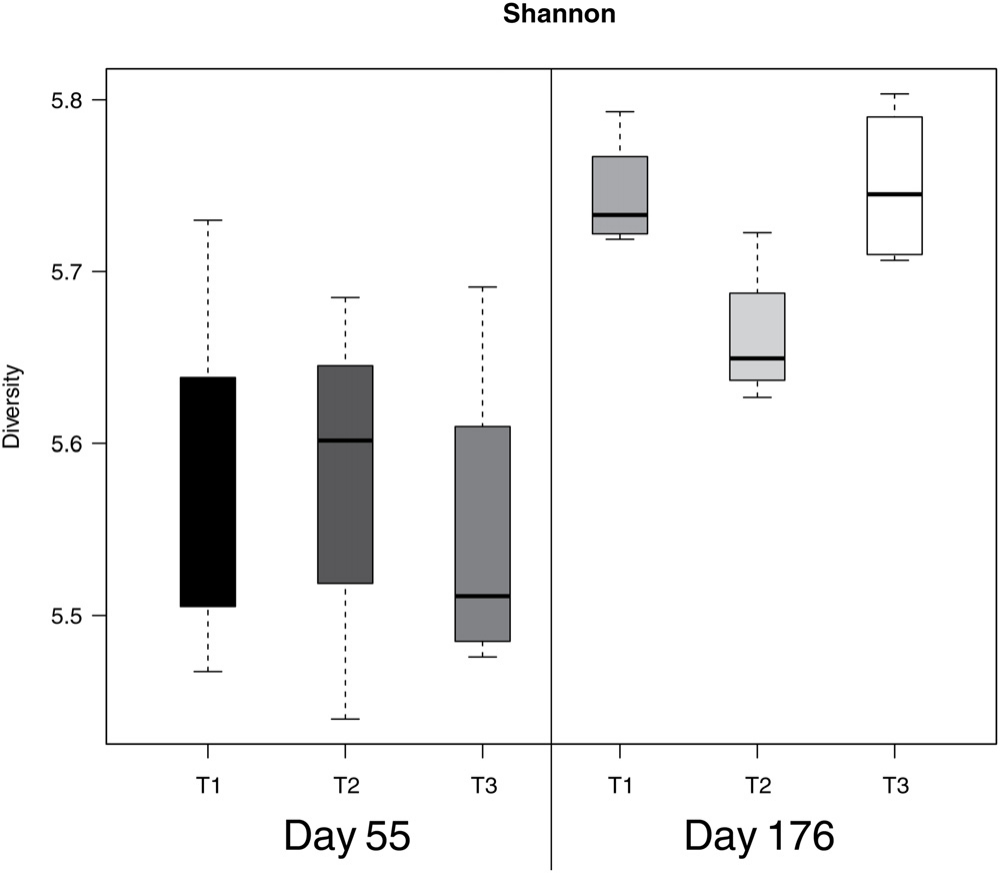
Diversity in microbiota in the three treatment groups on day 55 and 176. The Shannon index (y-axis) was calculated for all three treatments (T1, T2, and T3) on both days (55 and 176) (x-axis) based on probe-level data from the PITChip.

**Fig 3:**
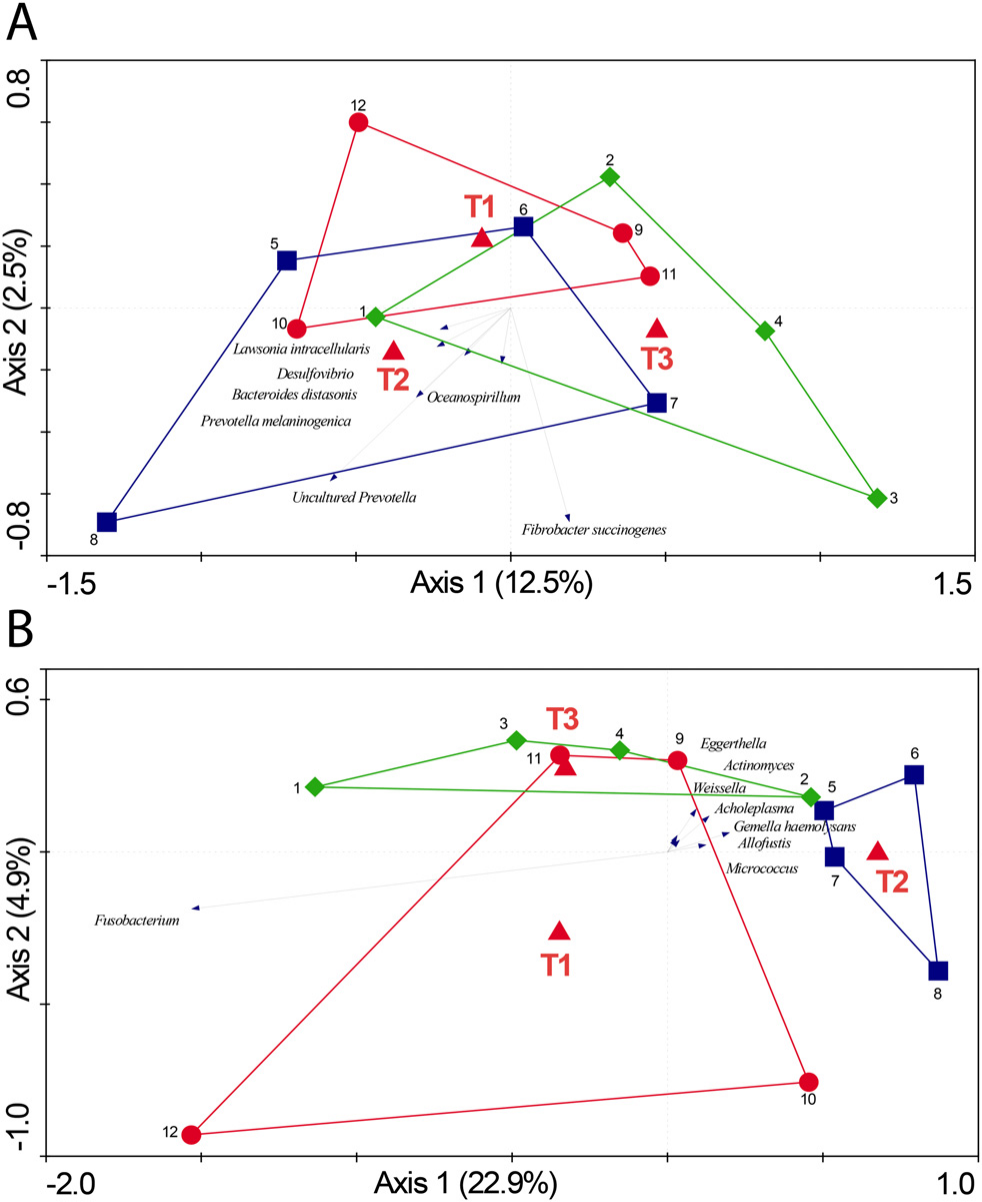
Triplot for RDA analysis of jejunal microbiota composition on day 55 and day 176. Nominal environmental variables T1, T2 and T3 are represented by red triangles (▲). Samples are grouped by treatment: T1 (red; ○), T2 (blue; □) and T3 (green; ◇), each symbol represents a pool of four pigs, and numbers represent pool identifiers. A) Top-panel shows the RDA analysis of jejunal microbiota composition on day 55. Microbial groups contributing at least 40% to the explanatory axes are represented as vectors. Both axes together explain 15% of the total variance in the dataset. B) Bottom-panel shows the RDA analysis of jejunal microbiota composition on day 55. Microbial groups contributing at least 52% to the explanatory axes are represented as vectors. Both axes together explain 27.8% of the total variance in the dataset.

To evaluate the impact of antibiotic treatment with and without different routinely used stressful management procedures on the composition of intestinal microbiota, the PITChip was used (S1 File). In 55 day old pigs no differences were found in the microbiota diversity (Shannon index based on probe-level profiles), however, in day 176 old pigs, the microbial diversity of antibiotic treated animals (T2) was significantly lower than for the other treatments (p<0.05) (Fig. 2). Multivariate redundancy analysis (RDA) of PITChip profiles at the approximate genus-level showed a high overlap of samples from different treatments on day 55 (Fig. 3A). The RDA diagram for day 176 showed that T2 animals could be distinguished from the other groups, and Monte Carlo Permutation testing showed T2 significantly contributed to explaining the observed variation in microbiota composition (p = 0.028) (Fig. 3B). In order to further investigate the changes in microbial groups between the treatments on each time-points, genus-level phylogenetic groups for which the relative abundance significantly differed between treatments were identified by univariate analysis, focusing on three separate comparisons, namely T2 vs. T1, T3 vs. T1, and T3 vs. T2 on both day 55 and day 176 separately (Table 1). This analysis revealed that on day 55 only two microbial groups differed significantly when comparing T3 and T2. At day 176 the relative abundance of multiple microbial groups was different, but only when comparing T3 or T1 versus T2 (for details see Table 1).

**Table 1:** Genus-level phylogenetic groups changed in T2 and/or T3 animals.

	T2 vs T1		T3 vs T1		T3 vs T2		ARC^1^		
Day 55	p^2^	q^3^	p	q	p	q	T1	T2	T3
*Coprococcus eutactus* et rel.	0.69	0.94	0.34	0.54	0.03↓	0.09	0.42±0.13	0.49±0.1	0.37±0.03
Uncultured *Prevotella*	0.20	0.34	0.69	0.94	0.03↓	0.09	0.34±0.17	0.58±0.25	0.24±0.05

Day 176	p	q	p	q	p	q	T1	T2	T3
*Actinomyces* et rel.	0.03↑	0.09	0.34	0.54	0.11	0.24	0.12±0.02	0.16±0.01	0.13±0.01
*Allofustis* et rel.	0.03↑	0.09	0.89	1.00	0.06	0.13	0.19±0.02	0.24±0.02	0.2±0.02
*Bacillus* et rel.	0.49	0.73	0.89	1.00	0.03↑	0.09	0.39±0.17	0.27±0.02	0.37±0.06
*Bacteroides distasonis* et rel.	0.11	0.24	1.00	1.00	0.03↑	0.09	0.51±0.31	0.15±0.04	0.52±0.24
*Bifidobacterium* et rel.	0.03↓	0.09	1.00	1.00	0.06	0.13	0.74±0.3	0.43±0.02	0.67±0.19
Eggerthella et rel.	0.03↑	0.09	0.06	0.13	0.20	0.34	0.11±0.01	0.13±0.01	0.12±0.01
*Fusobacterium* et rel.	0.03↓	0.09	0.89	1.00	0.03↑	0.09	0.62±0.35	0.16±0.03	0.56±0.24
*Neisseria* et rel.	0.03↓	0.09	1.00	1.00	0.06	0.13	0.3±0.11	0.17±0.01	0.28±0.08
*Oxalobacter* et rel.	0.03↓	0.09	0.20	0.34	0.20	0.34	0.34±0.11	0.2±0.02	0.25±0.05
*Prevotella melaninogenica* et rel.	0.06	0.13	1.00	1.00	0.03↑	0.09	0.21±0.12	0.08±0.01	0.2±0.09
*Ruminococcus ganvus* et rel.	0.34	0.54	1.00	1.00	0.03	0.09	0.15±0.03	0.17±0.01	0.14±0.02
*Streptococcus salivarius* et rel.	0.20	0.34	0.69	0.94	0.03↑	0.09	0.3±0.19	0.1±0.05	0.36±0.18
*Streptococcus suis* et rel.	0.11	0.24	0.69	0.94	0.03↑	0.09	2.6±1.67	0.68±0.4	3.06±1.62
*Sutterella wadsorthia* et rel.	0.03↓	0.09	0.49	0.73	0.20	0.34	0.3±0.05	0.23±0.02	0.28±0.07
Uncultured *Mollicutes*	0.06	0.13	1.00	1.00	0.03↓	0.09	0.52±0.06	0.6±0.05	0.52±0.03
Uncultured *Prevotella*	0.06	0.13	1.00	1.00	0.03↑	0.09	1.11±0.64	0.43±0.07	1.08±0.46

^1^ ARC: average relative contribution [%] of a microbial group. Values represent means ± SD. The microbial groups with a relative abundance lower than 0.1% in all three treatments are not shown.

^2^ “↑” or “↓” indicates whether the average relative contribution of the microbial group was increased or decreased in the particular comparison.

^3^q is the corrected p-value (Benjamini Hochberg)

### Transcriptomic analyses

**Fig 4:**
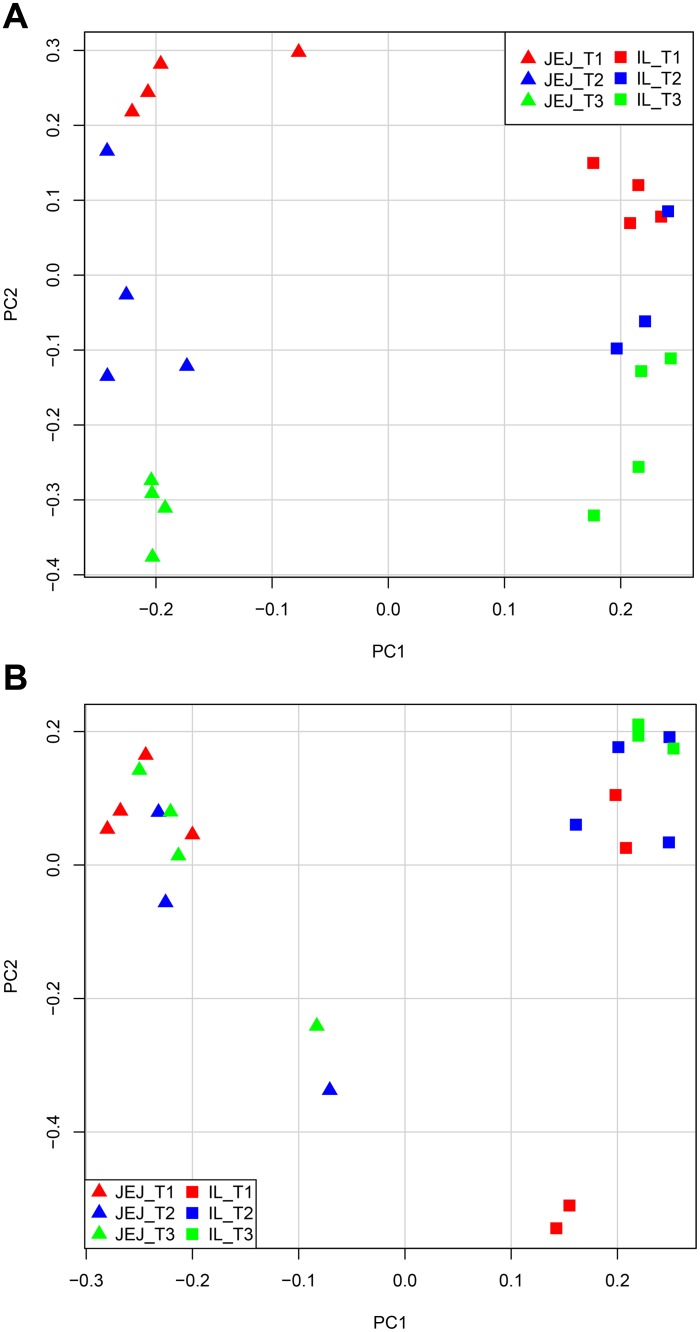
Principal Component Analysis of jejunal and ileal tissue gene expression for three different treatments at day 55 and 176. Each symbol represents all expressed genes (approximately 44k probes) of a particular sample. A) top-panel represents day 55 and B) bottom-panel represents day 176. Three different treatments are depicted, T1 (red), T2 (blue) and T3 (green) and two different tissues, jejunum (JEJ, triangles) and ileum (IL, squares).

To evaluate the impact of antibiotic treatment with and without different routinely used stressful management procedures on the intestinal tissue gene expression, the Agilent microarray was used. Principal Component Analysis (PCA) was performed to get more insight into the variability in the data. To this end, only the first and second principal components were taken into account for both time-point analyses, because they were found to explain the largest part of the variation in the data. The variance explained by the first two axes was 58.2% for day 55 and 45.8% for day 176. Data clustering occurred for similar treatments (Fig. 4A, red; T1, blue; T2, and green; T3), with a more clear distinction between the treated groups (T2 and T3) and the control group (T1). Furthermore, we observed a clear distinction between the two different tissues, jejunum and ileum, at both day 55 and 176 (Fig. 4B). At day 55 the tissues are separated by principal component 1 (PC1), and the different treatment groups are separated by principal component 2 (PC2). In contrast, at day 176, more overlap between treatment groups could be observed along PC2, due to a large variation within the treatment groups as compared to the variation between treatment groups, indicating that there are no significant differences in gene expression between the different treatments.

To investigate the effect of the three treatments on jejunum and ileum tissue gene expression in more detail, two approaches were performed 1) using a subset of genes that were found to be significantly differently expressed as based on ANOVA analysis, and 2) using all ‘expressed’ genes. An ANOVA analysis was performed on both day 55 and 176. All probes/genes which were significant under p_cor_ < 0.05 and absolute Fold Change > 1.5 in one of the six comparisons were taken for further functional and enrichment analyses (S1 Table). The significantly up- and down-regulated genes, p_cor_<0.05 and FC>|1.5|, were used as input for functional analyses. First, DAVID functional annotation clustering was performed resulting in multiple groups with a significant Enrichment Score (ES > 1.3), for jejunum and ileum at day 55 (see Tables 2–4 for top 5 results from the DAVID functional annotation clustering).

**Table 2:** Significantly enriched DAVID clusters (ES > 1.3) comparing T2 vs. T1 at day 55.

Tissue		Down		Up	
	**#**	**Generalized Term**	**ES**	**Generalized Term**	**ES**
**Jejunum**	1	x	x	vacuole/lysosome	4.1
	2	x	x	gene expression epigenetic / gene silencing	3
	3	x	x	nucleotide binding	2.9
	4	x	x	organelle lumen	2.7
	5	x	x	(positive) regulation of protein kinase activity (metabolic)	2.7
**Ileum**	1	protease inhibitor	1.48	TNF/cytokine activity	1.5
	2	Serine/threonine protein kinase	1.36	x	x

**Table 3:** Significant enriched DAVID clusters (ES > 1.3) comparing T3 vs. T1 at day 55.

Tissue		Down		Up	
	**#**	**Generalized Term**	**ES**	**Generalized Term**	**ES**
**Jejunum**	1	transcription / nucleotide metabolic process	1.57	nucleotide binding	7.22
	2	transcription regulation	1.45	vacuole/lysosome	4.95
	3	oxidative phosphorylation / electron transport	1.30	organelle lumen	4.57
	4	x	x	protein kinase activity	4.14
	5	x	x	protein localization / transport	3.81
**Ileum**	1	(sex) differentiation	1.72	tight junction/cell adhesion	2.26
	2	extracellular region	1.45	vesicle (cytoplasmic)	1.83
	3	x	x	Pleckstrin homology	1.63
	4	x	x	(positive) regulation of protein kinase activity	1.57
	5	x	x	regulation of cell migration /motility (leukocytes) / response to external stimulus	1.40

**Table 4:** Significant enriched DAVID clusters (ES > 1.3) comparing T3 vs. T2 at day 55.

Tissue		Down		Up	
	**#**	**Generalized Term**	**ES**	**Generalized Term**	**ES**
**Jejunum**	1	muscle tissue development	1.96	protein transport / localization	3.23
	2	transcription / nucleotide metabolic process	1.53	vacuole/lysosome	1.86
	3	metabolic process (vitamin/hormone)	1.47	x	x
	4	apoptosis	1.42	x	x
**Ileum**	1	regulation of apoptosis	1.76	organelle lumen	2.85
	2	axon/cell projection	1.73	regulation of cell migration / motility	2.80
	3	negative regulation apoptosis	1.52	SH3 domain	2.01
	4	x	x	vacuole/lysosome	1.97
	5	x	x	Lymphocyte/leukocyte homeostasis / apoptosis	1.94

In a second approach no pre-filtering of genes was performed, and all probes/genes were used as input to Gene Set Enrichment Analysis (GSEA). This resulted in the identification of similar biological processes affected by the treatments as observed by the DAVID analysis (S2 File). In contrast, when investigating day 176, no significantly enriched clusters were found in the DAVID analysis, however, the GSEA, in which all genes were used as input, revealed differences between the treatments in processes related to metabolism and immunity.

## Discussion

In this paper we provide evidence that a treatment at day four of life has long lasting effects on microbial composition and diversity, as well as on host gene expression in intestinal tissue. Due to the experimental design, all three groups were exposed to similar environmental conditions, that means that during this experiment similar exposure to pathogenic load, stress (besides treatment), as well as feed composition, condition and amount was encountered by these pigs, suggesting that the (clinical) history of these animals is almost identical. In a previous paper we reported on the short-term effects (eight days after birth) of an antibiotic and stress treatment which was administered at four days after birth as experienced by piglets under normal intensive husbandry conditions on the microbial colonization and immune development [16]. Here we show that the effects of these treatments can still be observed later in life, especially on day 55 after birth, supporting the idea that due to the used treatments, the animals ‘program’ their immune system differently at early age.

### Comparisons of microbiota composition and diversity between treatment groups

Based on the microbiota analyses, we conclude that there is an increase of diversity over time with an average of 5.48 at day 8 [16], 5.55 at day 55 and 5.75 at day 176, which is in agreement with similar observations in other pig studies [31,32]. In other words, the ecosystem becomes more diverse and presumably more stable in time. Conjointly the diversity and microbiota composition data suggest that the microbiota converge to an adult-type microbiota (more complex ecosystem), which is also reflected in the phylum level data (S2 Table). This conversion towards a ‘generic’ adult-type microbiota is strengthened by the experimental setup, because after weaning piglets were assigned to a pen which contained T1, T2 and T3 from the same and different litters. Interestingly, at day 176, we found a significant difference in microbiota diversity and composition of the pigs only treated with the antibiotic (T2) in comparison to the other treatment groups. Since all three groups were exposed to similar environmental conditions, the differences in microbiota diversity and composition observed at day 176 after birth can only be explained by group specific intrinsic animal factors. One could argue that the intestinal immune cells of the T2 animals were programmed differently from the T1 and T3 animals, due to the antibiotic treatment at day 4 after birth. Differences in immune programming may then lead to differences in immunologic and microbial homeostasis. Why these differences in microbial homeostasis cannot be observed at day 55 is not clear, but it could be explained by the fact that at day 55 the composition and diversity of the gut microbiota have not been stabilized yet due to the relative proximity to the weaning period, which occurred around day 26 in this study. The weaning period is known to cause a large shift in the composition and diversity of gut microbiota [33,34]. In this short time-period of weaning the gut ecosystem must progress from a rather simple stable equilibrium to a more complex stable equilibrium. Another explanation could be that in the life history between day 55 and 176 the T2 animals have reacted differently to an unknown external factor because the intestinal immune cells of the T2 animals were programmed differently from T1 and T3 animals, due to the antibiotic treatment at day 4 after birth. Because the clinical data (not shown) from this study did not suggest any signs of an infection, it is more likely due to the differences in immune programming that lead to differences in maintenance of immunologic and microbial homeostasis upon exposure to that presumed external factor. However, the results of more detailed analysis of the microbiota data are more supportive for our first explanation/hypothesis. When zooming in on the differences in microbial composition and diversity by the use of univariate analysis, we observed that two microbial groups differed significantly (p<0.05) when comparing antibiotic treated pigs with antibiotic and stress treated pigs, namely *Coprococcus eutactus* et rel. and uncultured *Prevotella* (Table 1). At day 176 a larger number of significant differences (p<0.05) in the relative abundance of genus-level microbial groups was found (8 groups each when comparing T2 versus T1 and T2 versus T3 pigs, respectively). It is striking that in T2 pigs almost in all cases the average relative contribution of these groups was lower compared to that found in T1 or T3 pigs. For example the following microbial groups were lower in T2, *Streptococcus suis* et rel., uncultured *Prevotella*, *Fusobacterium* et rel., *Bacteroides distasonis* et rel., and *Prevotella melaninogenica* et rel. (for details see Table 1). It has previously been shown that *Streptococcus suis*, a potential pathogen, is more abundant in the pig intestine directly after weaning [35]. Several species of *Prevotella*, including *Prevotella melaninogenica*, are also potential pathogens that mostly occur in the upper digestive tract [36,37]. *Bacteroides* spp. are in general mutualistic, and have been described to benefit their host by excluding potential pathogens from colonizing the gut [38]. However, other studies have also shown possible negative effects of increased *Bacteroides* spp. in the gut [39,40]. So all these bacteria described above have both beneficial and pathogenic characteristics. It should be noted, however, that in general, relative abundance of the phylum Bacteroidetes, encompassing both genera *Prevotella* and *Bacteroides*, was decreased in T2 animals at day 55, whereas it was increased at day 176 (S2 Table). Fusobacteria have a potent lipopolysaccharide (LPS), and are classified as pathogenic [41]. In conclusion the data presented here may indicate that the specific microbial composition in the T2 animals kept under the experimental conditions, i.e. antibiotics and environment (farm), is unfavourable for these microbial species.

### Comparison of gene expression in intestinal tissues between treatment groups

Whereas at day 176 no differential gene expression could be observed, the transcriptomics data showed differential gene expression at day 55, in both jejunum and ileum, which could be translated to (significant) differences in functional processes/pathways. To see which biological processes were affected by the treatment in these tissues, functional analyses were performed by DAVID analysis. This revealed higher expression of a number of processes, including immunological processes, in ileal tissue of T2 and T3 pigs compared to T1 pigs at day 55. In the ileum of T2 animals, the most prominent higher expressed immune-related process was; ‘TNF/cytokine activity’, and in T3 animals; ‘regulation of cell migration / motility (leukocytes) / response to external stimulus’, but also processes involved in intestinal barrier function (‘tight junctions/cell adhesion’). These data support our hypothesis that the programming of the immune system is altered due to the different treatments in early life, which is shown by the gene expression and/or microbiota changes of the intestinal system in the young adult (d55) and adult phase (d176) of life.

Despite the differences in gene expression at day 55 in both ileum and jejunum between the treatment groups, only the ileum displayed differences in immune related processes after DAVID analysis. This contrasts our previous data obtained for day 8 after birth that showed a major down-regulation of immune related processes in both jejunal and ileal tissue in the animals treated with antibiotic [16]. These observations are in agreement with the fact that differentiation of ileal and jejunal tissue occurs, and that in differentiated and matured ileum immunological structures like Peyer’s patches are much more abundant as compared to jejunum. To have a more complete picture of the different processes being affected by the treatments, we in addition performed functional Gene Set Enrichment Analysis (GSEA), and superimposed gene expression values over known (immunological) pathways for ‘adult’ life time-points. The GSEA results for day 55 were in concordance with the DAVID functional analyses. However, by taking into account the entire transcriptome, we observed that GSEA analyses showed additional enriched immunological processes compared to DAVID for T3 pigs.

To investigate whether the immune processes observed locally in ileal tissue at day 55 were also present systemically, we performed transcriptomic analyses on blood samples (data not shown). These blood transcriptomics data showed no dominance of immune processes in the different comparisons between treatments.

For 176 day old pigs, similar functional analyses were performed using DAVID and GSEA. However, the DAVID analyses did not result in any significantly enriched functional annotation clusters, which is likely due to the fact that almost no genes were significantly differently expressed between the treatments. In contrast, the GSEA for pigs of day 176 resulted in multiple significantly enriched gene sets, indicating that despite the more pronounced differences in microbial composition in adult life (176 vs. 55 days), host gene expression is not always changing accordingly. This might, in part, be explained by the fact that these pigs were ‘healthy’, and thus cells could simply perform ‘regular’, non immune-related functions. Nevertheless, in T1 and T3 pigs more enriched gene sets involved in immunity were observed compared to T2 pigs, suggesting that a higher microbial diversity in adult life is accompanied by more active genes involved in immunity, e.g. communication (cross-talk host-microbe), surveillance and response. However, these differences in gene set enrichment were only observed at the functional process level.

### Link between microbiota composition and tissue gene expression

The microbiota diversity and composition was highly similar at day 55 for all three treatments, whereas the gene expression showed markedly different processes when comparing the different treatment groups. This suggests, that the observed gene expression differences were due to the altered immune programming in early life. Interestingly, it was recently found that transient differences in piglets’ gut microbiota caused by amoxicillin treatment of sows before and after birth was associated with differences in intestinal physiology not only early in life, but also in adult pigs [17]. It is known that the microbiota have an important role in the development of (intestinal) immunity in the neonatal period [42]. Germ-free mice, for example, do not develop a proper working immune system because they lack microbial stimuli in early life [43]. Indeed, at day 8 early life environmental factors influenced the microbial colonization of these piglets, reflected in differences in microbial diversity and composition [16]. Thus altering the composition and/or diversity of microbiota in early-life by antibiotic treatment, or antibiotic treatment in combination with stressors, influences the programming of the (intestinal) immune development and consequently the immune homeostasis later in life. This alternate programming of the immune system, due to early life environmental factors, could have an impact on the (intestinal) health status of these pigs. In human studies it has already been shown that different early life environmental factors can lead to a higher risk of diseases such as asthma and allergy [44–46]. We did not subject these pigs to a pathogenic challenge, for example enterotoxigenic *Escherichia coli*, to test the putative differences in immune response between the different treatments groups in the adult stage.

**Fig 5:**
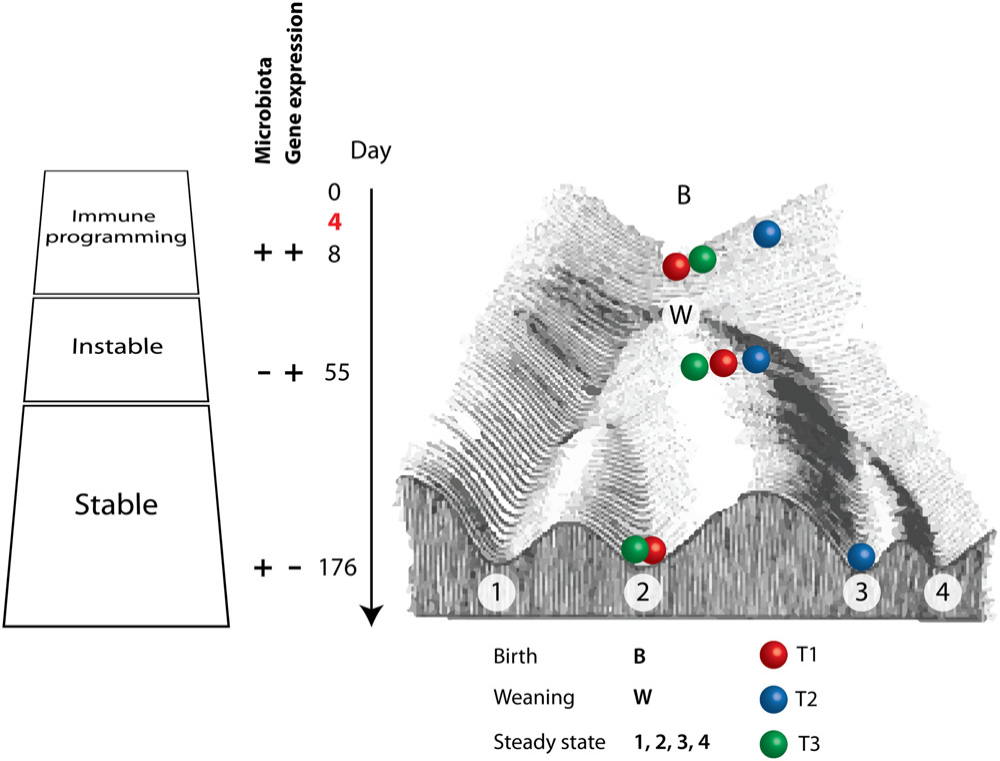
Schematic representation of results. Overview of the time-line, birth (day 0), administration of the treatments (day 4), measurements days 8, 55, and 176, as well as the hypothetical interpretation of all results from the whole experiment, results from the previous paper about day 8 are included too [16]. On the left we categorized the gut system in three different ‘blocks’, immune programming which occurs in early life, followed by an instable period which includes weaning and later in life a stable period (homeostasis). Note that the treatments, antibiotic and/or stress, were at day 4 during the immune programming period. Next to this the significant findings of microbiota or gene expression data between treatments per time-point are depicted by “+”, and no differences between treatments with a “-”. On the right a metaphorical landscape of the gut system in time is depicted, where the top is day 0 (birth) and bottom is day 176 (slaughter). Spheres depict the current state of the system for day 8, 55, and 176, and colours correspond to the different treatments (T1; red, T2; blue, and T3; green).

The gut is a complex (eco)system, where many interactions between host, microbiota and environment occur simultaneously. The state of the system is thus determined by internal and external conditions, and all these conditions are dynamic. If there would be a single steady state in this system, essentially after each perturbation the system would return to the same steady state. However, one could envisage that the gut has multiple alternative stable states, and that sufficiently severe perturbations to the system may lead to other steady states of the system. This way of thinking was visualized by Scheffer *et al*. [47] by generating stability landscape(s), where hills depict unstable states and valleys steady states. Deeper valleys are more resilient to perturbations, in other words more ‘energy’ is needed to push the system out of the steady state. The occurrence of several stable states has recently been confirmed for the human intestinal microbiota by the discovery of bimodal distributions of a number of microbial groups, the abundance of which is associated with host factors such as ageing and overweight [48]. Our hypothesis is that weaning (d28) of the piglets introduces a ‘newly’ acquired microbial community and is followed by an instable period, because of the introduction of solid feed and separation from the sow. This leads to high variance between individuals and could eclipse the differences between the treatment groups regarding the microbiota analyses. However, because the immune programming at young age was found significantly different between the treatment groups [16], the system still responds differently towards this ‘newly’ acquired microbial community. After weaning, during this instable period, the system will advance towards a stable state (homeostasis). Yet, the difference in immune programming between the gut systems, due to the treatments, enforce the systems to pursue different paths to reach a steady state (homeostasis) (Fig. 5). In these different steady states, the microbiota diversity and composition is different. However, this does not result in differential gene expression in the host intestinal tissue, because the system has adapted by constant cross-talk between host and microbiota to the new steady state.

### Conclusion

The use of antibiotics in combination with or without stressors in early life (day 4) affects ‘adult’ animals, both with respect to their microbiota as well as intestinal gene expression, such as differences in immunological processes displayed by ileal tissue. These differences on transcriptomic level are likely due to microbiota-induced differences in immune programming during the neonatal period, opening avenues to steer these and other processes through modulation of the microbial colonization process in the early days of an animal’s life.

## Supporting Information

S1 File Normalized microbiota data of jejunum on days 55 and 176 on probe, phylum, class, and genus/species level. (XLSX)

S2 File Results of Gene Set Enrichment Analysis (GSEA) comparing treatment groups at day 55 and 176. (XLSX)

S1 Table Overview of probes, annotated genes and DAVID identifiers significantly different between different treatments in both tissues at day 55 and day 176. (DOCX)

S2 Table Average relative abundance of phylum level microbial groups for each treatment at day 55 and 176. (DOCX)
