# Supplementary material for: Long-Lasting Effects of Early-Life Antibiotic Treatment and Routine Animal Handling on Gut Microbiota Composition and Immune System in Pigs
Source: PLoS One. 2015 Feb 6;10(2):e0116523. doi: 10.1371/journal.pone.0116523 (PMC4319779; doi:10.1371/journal.pone.0116523)
Supplement: S1 Table — (DOCX) [file pone.0116523.s003.docx]

**Supplemental Table S1** Overview of probes, annotated genes and DAVID identifiers significantly different between different treatments in both tissues at day 55 and day 176

| **Day** | **Tissue** | **Comparison** | **Regulation** | **Probes^a^** | **Ann. genes^a,b^** | **DAVID IDs** |
| --- | --- | --- | --- | --- | --- | --- |
| 55 | Jejunum | T2 vs. T1 | Down | 182 | 26 | 14 |
|  |  |  | Up | 823 | 479 | 410 |
|  |  | T3 vs. T1 | Down | 1042 | 138 | 99 |
|  |  |  | Up | 1277 | 803 | 700 |
|  |  | T3 vs. T2 | Down | 701 | 97 | 78 |
|  |  |  | Up | 296 | 151 | 125 |
|  | Ileum | T2 vs. T1 | Down | 306 | 55 | 41 |
|  |  |  | Up | 315 | 118 | 101 |
|  |  | T3 vs. T1 | Down | 663 | 48 | 36 |
|  |  |  | Up | 725 | 182 | 110 |
|  |  | T3 vs. T2 | Down | 371 | 50 | 39 |
|  |  |  | Up | 284 | 182 | 155 |
| **Day** | **Tissue** | **Comparison** | **Regulation** | **Probes** | **Ann. genes** | **DAVID** |
| 176 | Jejunum | T2 vs. T1 | Down | 19 | 3 | 2 |
|  |  |  | Up | 6 | 0 | 0 |
|  |  | T3 vs. T1 | Down | 6 | 1 | 1 |
|  |  |  | Up | 1 | 0 | 0 |
|  |  | T3 vs. T2 | Down | 6 | 0 | 0 |
|  |  |  | Up | 19 | 3 | 2 |
|  | Ileum | T2 vs. T1 | Down | 1 | 0 | 0 |
|  |  |  | Up | 4 | 0 | 0 |
|  |  | T3 vs. T1 | Down | 3 | 0 | 0 |
|  |  |  | Up | 4 | 0 | 0 |
|  |  | T3 vs. T2 | Down | 0 | 0 | 0 |
|  |  |  | Up | 0 | 0 | 0 |

^a^ p_cor_ < 0.05 and log Fold Change > |1.5|
^b^ Ann. Genes, annotated genes which have a gene symbol
Abbreviations used: T1; Treatment 1,T2; Treatment 2, T3; Treatment 3.
