# Supplementary material for: Long-Lasting Effects of Early-Life Antibiotic Treatment and Routine Animal Handling on Gut Microbiota Composition and Immune System in Pigs
Source: PLoS One. 2015 Feb 6;10(2):e0116523. doi: 10.1371/journal.pone.0116523 (PMC4319779; doi:10.1371/journal.pone.0116523)
Supplement: S2 Table — (DOCX) [file pone.0116523.s004.docx]

**Supplemental Table S2** Average relative abundance of phylum level microbial groups for each treatment at day 55 and 176

|  | **Day 55** | | | **Day 176** | | |  |
| --- | --- | --- | --- | --- | --- | --- | --- |
|  | T1 | T2 | T3 | T1 | T2 | T3 | |
| Firmicutes | 76.97±3.8^a^ | 79.01±2 | 78.2±1.49 | 79.15±3.75 | 79.73±3.11 | 80.81±3.62 | |
| Proteobacteria | 12.8±2.23 | 12.44±1.5 | 11.93±0.91 | 12.33±2.28 | 11.81±2.64 | 11.06±3.05 | |
| Bacteroidetes | 3.46±1.16 | 2.37±0.34 | 3.49±0.74 | 1.95±0.36 | 2.47±0.42 | 2.19±0.66 | |
| Spirochaetes | 2.67±0.3 | 2.87±0.2 | 2.49±0.3 | 2.62±0.34 | 2.42±0.8 | 2.72±0.41 | |
| Actinobacteria | 2.54±0.6 | 2.41±0.11 | 2.4±0.27 | 3.2±1.48 | 2.63±0.5 | 2.33±0.72 | |
| Fibrobacteres | 0.5±0.19 | 0.27±0.08 | 0.52±0.18 | 0.23±0.08 | 0.34±0.06 | 0.41±0.13 | |
| Fusobacteria | 0.62±0.35 | 0.16±0.03 | 0.56±0.24 | 0.14±0.04 | 0.2±0.08 | 0.11±0.05 | |
| Deferribacteres | 0.23±0.04 | 0.24±0.04 | 0.21±0.02 | 0.2±0.03 | 0.22±0.07 | 0.19±0.07 | |
| Verrucomicrobia | 0.21±0.04 | 0.22±0.03 | 0.19±0.02 | 0.17±0.02 | 0.19±0.06 | 0.17±0.06 | |
| Chlamydiae | 0±0 | 0±0 | 0±0 | 0±0 | 0±0 | 0±0 | |
| Planctomycetes | 0±0 | 0±0 | 0±0 | 0±0 | 0±0 | 0±0 | |

^a^ The average relative contribution [%] of a microbial group is depicted with their respective standard deviation

Abbreviations used: T1; Treatment 1,T2; Treatment 2, T3; Treatment 3.
